# Enhanced release of acid sphingomyelinase-enriched exosomes generates a lipidomics signature in CSF of Multiple Sclerosis patients

**DOI:** 10.1038/s41598-018-21497-5

**Published:** 2018-02-15

**Authors:** Damiana Pieragostino, Ilaria Cicalini, Paola Lanuti, Eva Ercolino, Maria di Ioia, Mirco Zucchelli, Romina Zappacosta, Sebastiano Miscia, Marco Marchisio, Paolo Sacchetta, Marco Onofrj, Piero Del Boccio

**Affiliations:** 1 Department of Medical, Oral and Biotechnological Sciences, University “G. d’Annunzio” of Chieti-Pescara, Chieti, Italy; 2 Centre on Aging Sciences and Translational Medicine (Ce.S.I-MeT), University “G. d’Annunzio” of Chieti-Pescara, Chieti, Italy; 3 Department of Pharmacy, University “G. d’Annunzio” of Chieti-Pescara, Chieti, Italy; 4 Department of Medicine and Aging Sciences, “G. d’Annunzio” University of Chieti-Pescara, Chieti, Italy; 5 Department of Neuroscience, Imaging and Clinical Sciences, “G. d’Annunzio” University of Chieti-Pescara, Chieti, Italy

## Abstract

Multiple Sclerosis (MuS) is a complex multifactorial neuropathology, resulting in heterogeneous clinical presentation. A very active MuS research field concerns the discovery of biomarkers helpful to make an early and definite diagnosis. The sphingomyelin pathway has emerged as a molecular mechanism involved in MuS, since high levels of ceramides in cerebrospinal fluid (CSF) were related to axonal damage and neuronal dysfunction. Ceramides are the hydrolysis products of sphingomyelins through a reaction catalyzed by a family of enzymes named sphingomyelinases, which were recently related to myelin repair in MuS. Here, using a lipidomic approach, we observed low levels of several sphingomyelins in CSF of MuS patients compared to other inflammatory and non-inflammatory, central or peripheral neurological diseases. Starting by this result, we investigated the sphingomyelinase activity in CSF, showing a significantly higher enzyme activity in MuS. In support of these results we found high number of total exosomes in CSF of MuS patients and a high number of acid sphingomyelinase-enriched exosomes correlated to enzymatic activity and to disease severity. These data are of diagnostic relevance and show, for the first time, high number of acid sphingomyelinase-enriched exosomes in MuS, opening a new window for therapeutic approaches/targets in the treatment of MuS.

## Introduction

Multiple Sclerosis (MuS) is a highly complex CNS disease characterized by a multifactorial pathogenesis and a great heterogeneity in clinical presentation^1,2^. A very active MuS research field concerns the discovery of new biomarkers helpful to understand its etiology, and to assist in early diagnosis as well as prognosis^3^. CSF is an excellent source of biological information regarding the CNS, since it is in close contact and accurately reflects alterations of this system. Since the brain is the organ with one of the highest lipid concentration, lipid metabolism become of specific interest in understanding the molecular mechanism underlined to different neurological disease^4^, including MuS^5^. Sphingolipids are integral components of biomembranes, which are involved in many cellular functions, including cell proliferation, signaling cascades and apoptosis^6^. Sphingolipids include sphingomyelins (SMs), ceramides and sphingosines, involved in the same molecular cascade, in which one component emerges from the other by sequential enzymatic reactions^7^. The interest in the study of the SMs pathway increased with the use of FTY720 as an oral drug in MuS treatment. Indeed, the drug is an analogue of Sphingosine1-P, that represents the last product of SMs cascade^8^. In addition, higher phospholipid and lower sphingolipid content were found in white and gray matter from MuS patients compared to controls^9^, while sphingosine content is increased in MuS white matter^10^. On the other hand, ceramide levels increase in regions surrounding plaques^11^, and it was recently demonstrated, through a lipidomics approach, that patients with MuS had increased CSF levels of ceramide C16:0 and C24:0. Moreover, the presence of these ceramide species are sufficient to induce neuronal mitochondrial dysfunction and axonal damage^12^. Ceramides are the hydrolysis product of SMs through a reaction catalyzed by sphingomyelinase (SMase), a family of enzymes that have recently become of interest in MuS. Neutral SMase (nSMase) was also identified as a possible biomarker of MuS^13^, while, Acid SMase (ASMase) deficiency seems to enhance myelin repair after acute and chronic demyelination^14^. The exact mechanism of SMase release and activity (strongly dependent on the pH of environment) are still unclear, although the constitutive extracellular release, through exocytosis mechanism, was described and seems to be linked to exosomes and microvesicles (MVs) formation^15,16^. However, the role of exosomes and microvesicles in the release of secretory SMase and their involvement in MuS remain unclear^17^, even if some evidence is beginning to emerge^18^. Moreover, the link between SMs and vesicles was already demonstrated since the FTY720 significantly reduced the amount of MVs in the CSF of EAE-treated mice^19^. In our previous studies, we reported lipidomics profiling of serum^20^ and CSF^21^ from MuS patients. In the present work, we applied a lipidomics approach for investigating CSF phospholipid profile, with particular focus on SMs, in patients with MuS compared with CSF from patients suffering from Other Central and Peripheral Neurological Disease (C_OND and P_OND, respectively). Moreover, the ASMase activity in CSF of MuS patients and patients with C_OND and P_OND was measured and correlated to the number of total exosomes in CSF. Finally, CSF exosomes carrying ASMase were identified, gated and counted in the enrolled patients.

## Results

### Lipidomics by LC-ESI-MS/MS

**Figure 1:**
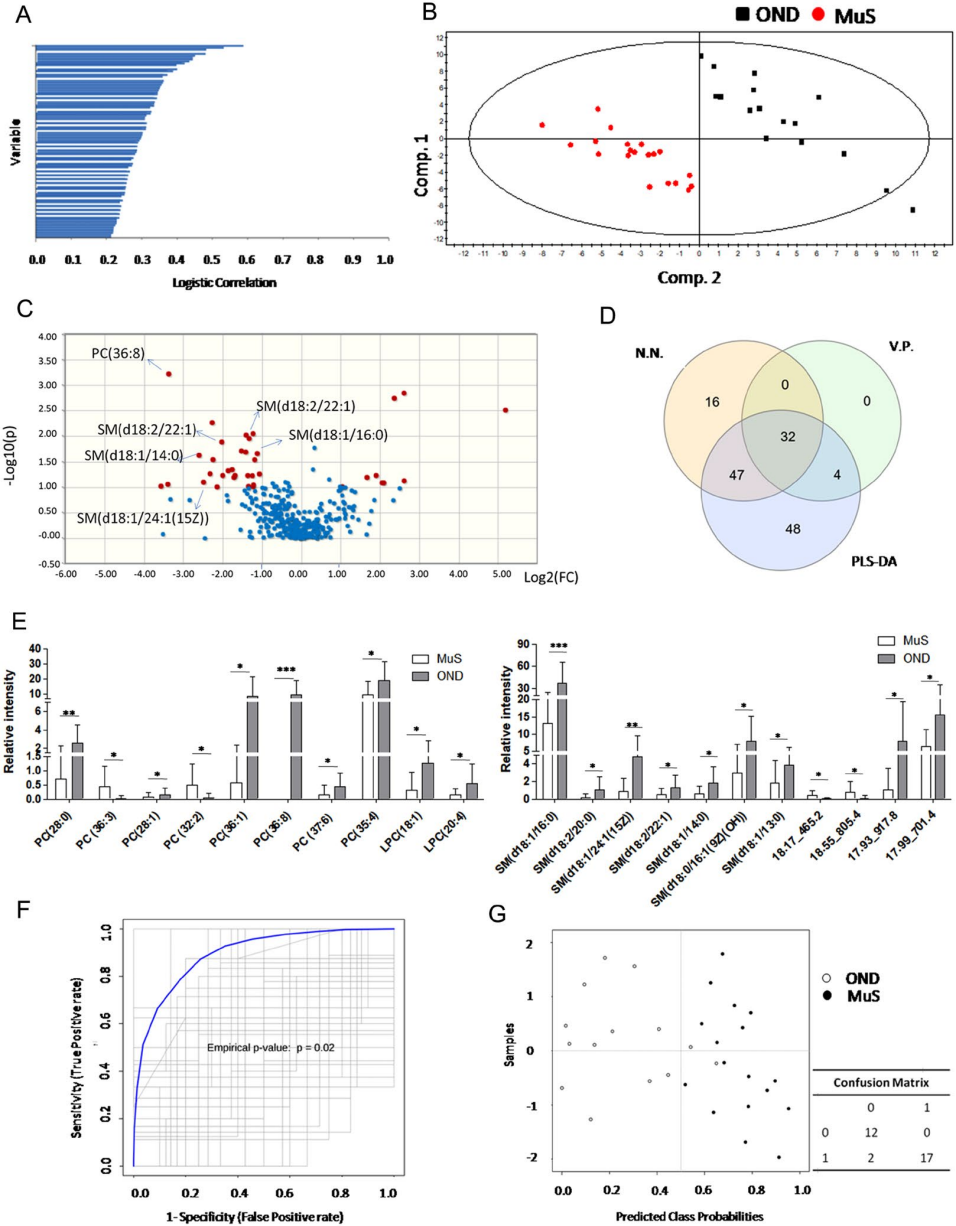
(**A**) The logistic correlation with the target (diagnosis) obtained by neural network implementation on 421 lipid signals, highlighting 96 lipid signals (inputs). The logistic correlation is reported as a numeric value between 0 and 1 that expresses the strength of the relationship between a single input and output variables. (**B**) Partial Last Square Discriminant Analysis (PLS-DA) scores scatter plot calculated on three components by using the 421 variables. The supervised multivariate analysis shows R2Y = 0.98, while Q2(cum) = 0.463. The PLS-DA shows unambiguous separation between the two clinical groups (black dots for OND patients and red dots for MuS). (**C**) Volcano Plot built on 421 Variables. The volcano plot is a combination of fold change (FC) and t-tests. The x-axis is log2 FC and the Y-axis is log10 (p-value) obtained by non-parametric test and by assuming unequal variance for both classes. The light blue dots are the non-significant variables and the red dots are significant with the −log10 (p-value) > 1. (**D**) Venn diagram based on the 96 lipid signals obtained by Neural Network (N.N), 131 signals obtained by PLS-DA and 36 variables obtained by Volcano plot (V.P.). (**E**) Histograms reporting the relative intensity of the most significant lipid species in MuS and OND CSF. *Means p < 0.05, **means p < 0.001 and ***means p < 0.0001 obtained by Mann-Whitney test. Sphingomyelins and Phosphatidylcholines are reported according their Systematic Names. (**F**) Combine ROC curve of the selected lipids for the evaluation of predictive accuracy of the model, using 100 permutations tests. The plot shows the AUC of all permutation, highlighting the actual observed AUC (0.942) and the empirical p value (0.02). (**G**) Plot of the predicted class probabilities of each sample through the 100-cross validation, underlying good predictivity of the proposed model in discriminating MuS patients from OND. Details of the reclassification are reported in the confusion matrix associated (0 means OND and 1 means MuS).

CSF lipids from 20 MuS patients and 17 OND patients were analyzed by our already reported method^22^ focusing on the acquisition of phosphatidylcholines (PCs) and SMs profile. 421 signals of PCs and SMs species were obtained following the data processing described in method section, and their relative intensities were subjected to different explorative statistical approaches with the aim of highlighting specific altered lipid patterns in MuS. Figure 1A shows the logistic correlation with the target (diagnosis) obtained by neural network implementation on data matrix, highlighting 96 lipid signals (inputs). The final architecture of the neural network obtained with 3 hidden neurons and its internal validation were reported in supplementary materials Fig. S1 and Table S1, respectively.

Figure 1B displays the Partial Least Squares-Discriminant Analysis (PLS-DA) score scatter plot obtained by using the 421 variables and shows an unambiguous separation between the two clinical groups analyzed (black dots for OND patients and red dots for MuS subjects). The PLS-DA analysis shows R2Y = 0.98, while Q2(cum) = 0.463 with 3 components. A number of 131 lipid signals were selected as significant with Variable Important in the Projection (VIP) score >1.0.

Finally, we used a more stringent univariate statistical analysis comparing all the 421 lipid signals taken individually. Figure 1C shows the results of univariate approach performed by using the Volcano Plot on all the PCs and SMs species. Thirty-six lipid signals are resulted discriminant metabolites between MuS and OND CSF samples. By comparing the results obtained through the three explorative approaches, thirty-two discriminant variables are resulted common, as reported in Venn diagram of Fig. 1D. These lipid signals were tentatively identified by Lipidmaps/Human Metabolome (HMDB) Databases search, by the retention time and by using the mass fragmentation data, obtaining 21 putative species (4 of which unidentified). The histograms in Fig. 1E show the relative intensity of putative lipid species significantly regulated in MuS. Interestingly, the most discriminating lipids appear to be less concentrated in MuS and, in particular, all the identified SMs showed this trend.

In order to evaluate the diagnostic predictive power of the selected lipids, we performed a combined Receiver Operating Characteristic (ROC) curve in a training set of patients (80%), as well as for external validation purpose, we predict diagnosis for new samples (testing set, 20%). In Fig. 1F was reported the plot of the 100 permutations tests using the Area Under the Curve (AUC) of the model as a measure of performance. The plot shows the AUC of all permutation, highlighting the actual observed AUC (0.942) in blu, along with showing the empirical p value (0.02). In addition, Fig. 1G shows the predicted class probabilities of each sample through the 100-cross validation, underlying good predictivity of the proposed model in discriminating MuS patients from OND (as reported in the confusion matrix associated). As external validation of the model we performed a new sample prediction analysis in the testing set of patients, showing that five patients out of six were correctly classified as reported in the Table S2 in Supplementary materials. Our lipidomics data indicate that these lipids can be used and studied as potential biomarkers of disease. As example of our lipidomics data application, we selected a sub-group of more significant SMs to build a diagnostic function to patients classification. The logistic regression model and its performance details are reported in Tables S3 and S4 in Supplementary Materials.

### Acid sphingomyelinase expression and activity in CSF

**Figure 2:**
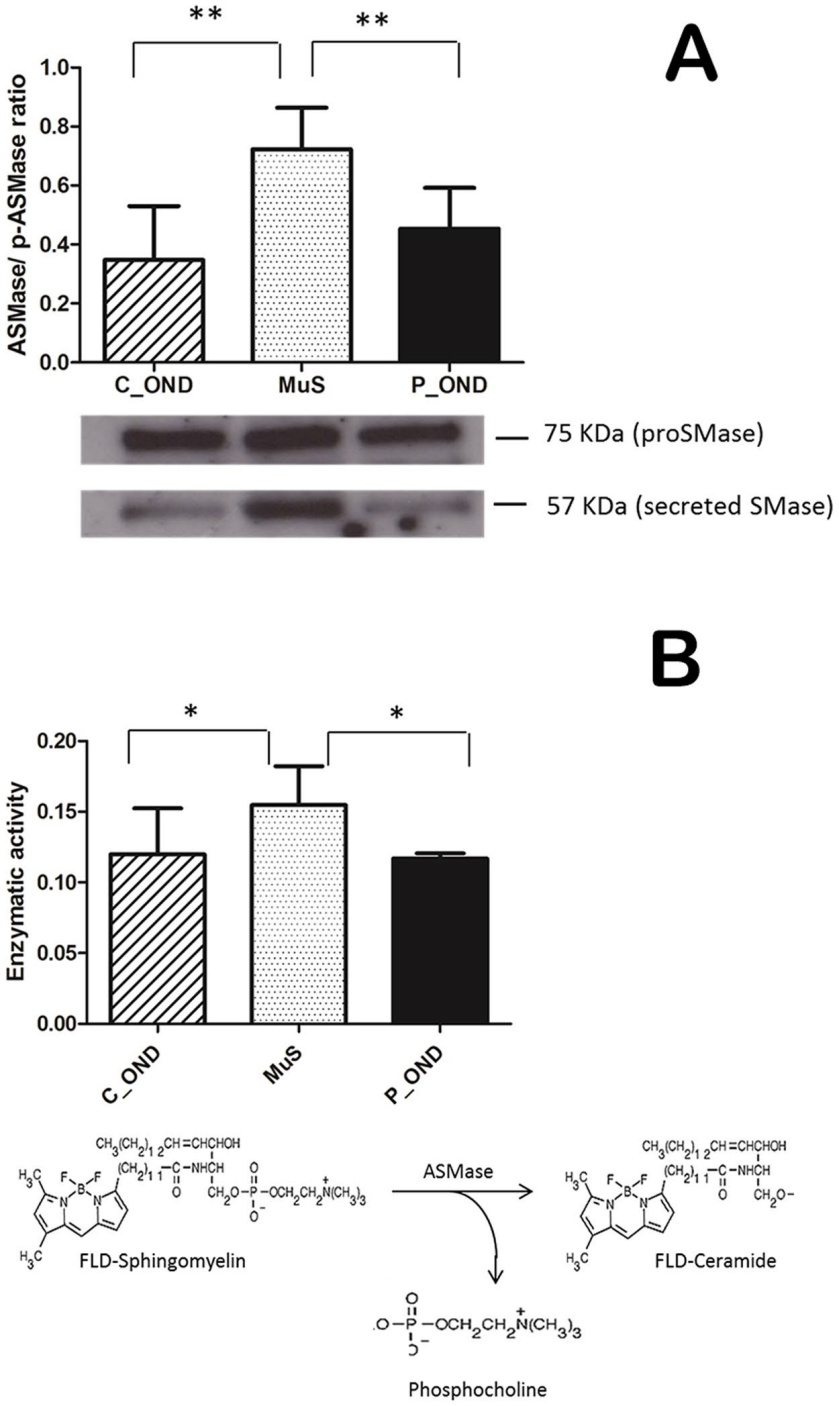
(**A**) ASMase expression in pooled CSF from MuS patients vs pooled OND CSF (divided in central and peripheral disease). The histograms show the mean normalized OD of the ASMase band (57KDa) relative to the OD of the proASMase band (75KDa). Error bars show the standard error of the mean from three independent pools of each disease condition. The image of WB is cropped from the same gel reported in Fig. S7. (**B**) Enzymatic activity of ASMase in CSF of the same pooled samples by using fluorescent SM-FLBODIPY (reaction reported in the scheme) incubated with CSF and analyzed by HPLC-FLD. C_OND and P_OND mean Other Central and Peripheral Neurological Diseases, respectively. **Means p < 0.001 obtained by Mann-Whitney test.

ASMase is a key enzyme in sphingolipid metabolism and it is involved in the regulation of cell fate and signaling via hydrolysis of sphingomyelin to ceramide. Recently, an involvement of this enzyme in MuS and in particular in demyelination mechanism was established^6,14^. These studies are in agreement with our lipidomics results where the low levels of several SMs in CSF of MuS patients means an altered sphingolipid metabolism. To elucidate the potential involvement of ASMase in MuS, we analyzed the expression and the activity of secretory isoforms^16^ of ASMase in CSF. Figure 2A shows the expression of ASMase isoforms at 57 kDa and at 75 kDa (named proSMase) in three independent pooled CSF from MuS patients (five patients for each pool) vs CSF from three pooled central OND (C_OND) and three pooled peripheral OND (P_OND). A significant increase in ASMase expression in MuS patients (p < 0.001, Mann-Whitney test) was detected, but no differences in expression resulted by the proSMase isoform at 75 kDa. We used a modified published method^23^ for measuring ASMase activity in human CSF (of the same pooled samples) to determine if the overexpression of the protein was also correlated to an increase of enzymatic activity. As shown in Fig. 2B, the ASMase was significantly more active in MuS than in both OND groups analyzed (p < 0.05). We measured the activity of neutral isoform of SMase in CSF. Our results, reported in Fig. S2, showed no activity of nSMase in all CSF analyzed, suggesting that the balance between SM and ceramide levels in CSF was dependent exclusively by acid isoform of the enzyme.

### Acid sphingomyelinase activity in CSF as a biomarker of Multiple Sclerosis

**Figure 3:**
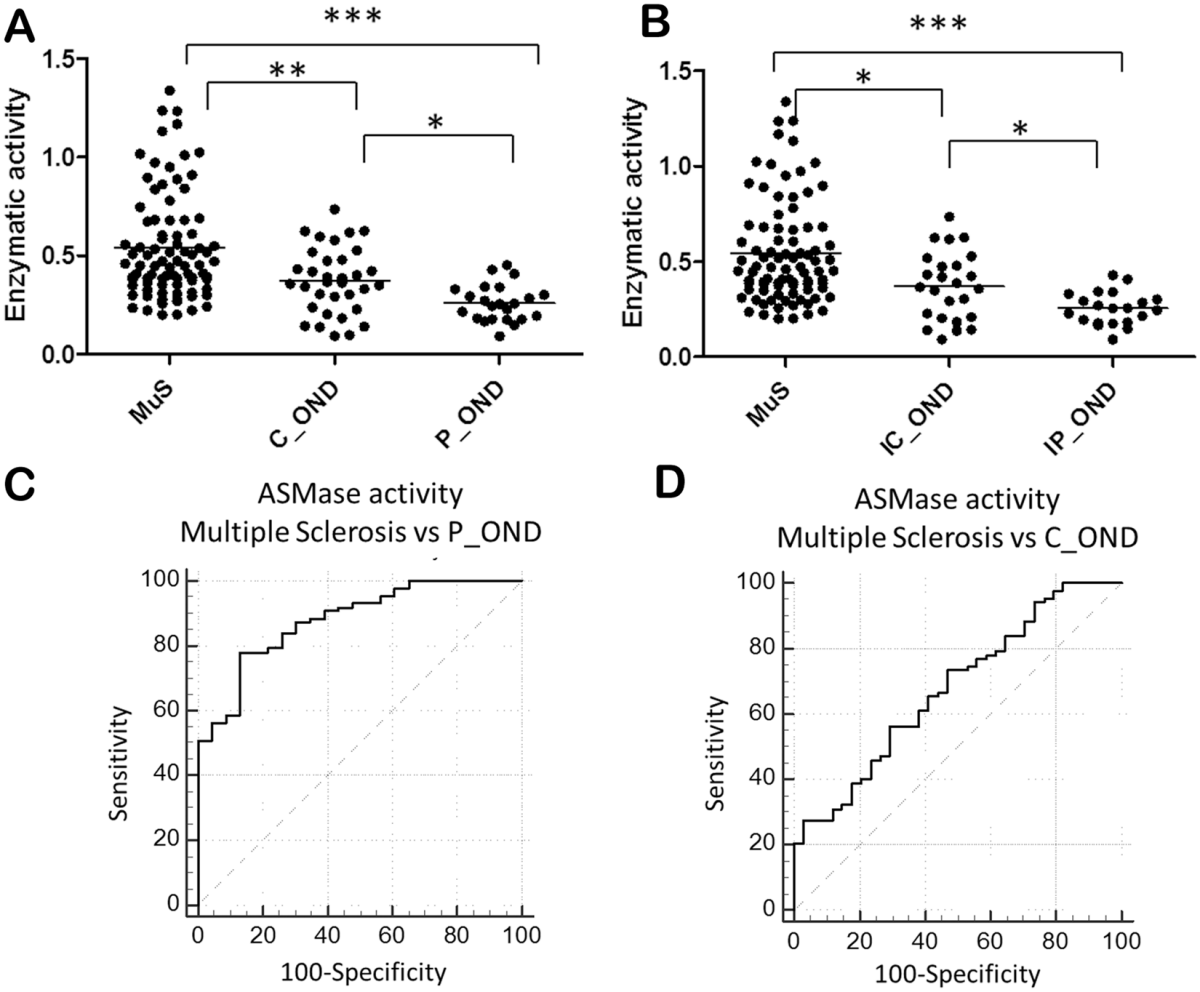
(**A**) CSF ASMase activity calculated in 87 MuS CSF and 57 OND patients divided into 34 C_OND and 23 P_OND. (**B**) Comparison of CSF ASMase activity in MuS patients and a sub-group of inflammatory neurological diseases, obtained by the exclusion of non-inflammatory diseases from the OND data set. (**C**) Receiver Operating Characteristic (ROC) curve for ASMase activity in MuS patients vs P_OND obtaining (p < 0.0001) an Area Under the Curve (AUC) of 0.88, sensitivity = 78.16% and specificity = 86.96% for a cutoff = 0.34. (**D**) ROC curve for ASMase activity in MuS patients vs C_OND obtaining (p < 0.001) AUC = 0.68, sensitivity = 56.32% and specificity = 70.59% for a cutoff = 0.43. *Means p < 0.05, **means p < 0.001 and ***means p < 0.0001 of Kruskal-Wallis test with Dunn’s Multiple Comparison post-test.

In order to investigate whether the measurement of ASMase activity may represent a potential diagnostic tool for MuS, we analyzed 87 MuS CSF and 57 OND patients divided into 34 C_OND and 23 P_OND. Figure 3A shows the ASMase activity in the CSF of the enrolled patients, showing a significantly higher activity (p < 0.0001 for the Kruskal-Wallis test) in CSF of MuS patients compared to both OND groups obtained by Dunn’s multiple comparison post-test (p < 0.0001 for P_OND and p < 0.001 for C_OND).

Similar results were achieved by comparing MuS with a sub-group of inflammatory neurological diseases, suggesting that ASMase behavior in CSF is independent of unspecific inflammatory condition (Fig. 3B). A pearson correlation test was performed to determine if ASMase activity was age dependent, showing no significant correlation (p = 0.13, R^2^ = 0.03) as reported in Fig. S3. In Fig. 3 panels C-D are reported the ROC curves based on this activity data, showing an AUC of 0.88 (p < 0.0001), with a sensitivity of 78.16% and specificity of 86,96% in distinguishing MuS patients from P_OND through a cut-off of 0.34. Whereas, the threshold that best distinguished MuS from C_OND patients was set to 0.43, resulting an AUC = 0.68 (p < 0.001), with sensitivity of 56.32% and specificity of 70.59%. More details of the performed ROC curves are reported in Supplementary Table S5.

### Acid sphingomyelinase activity correlates with the number of exosomes in CSF

**Figure 4:**
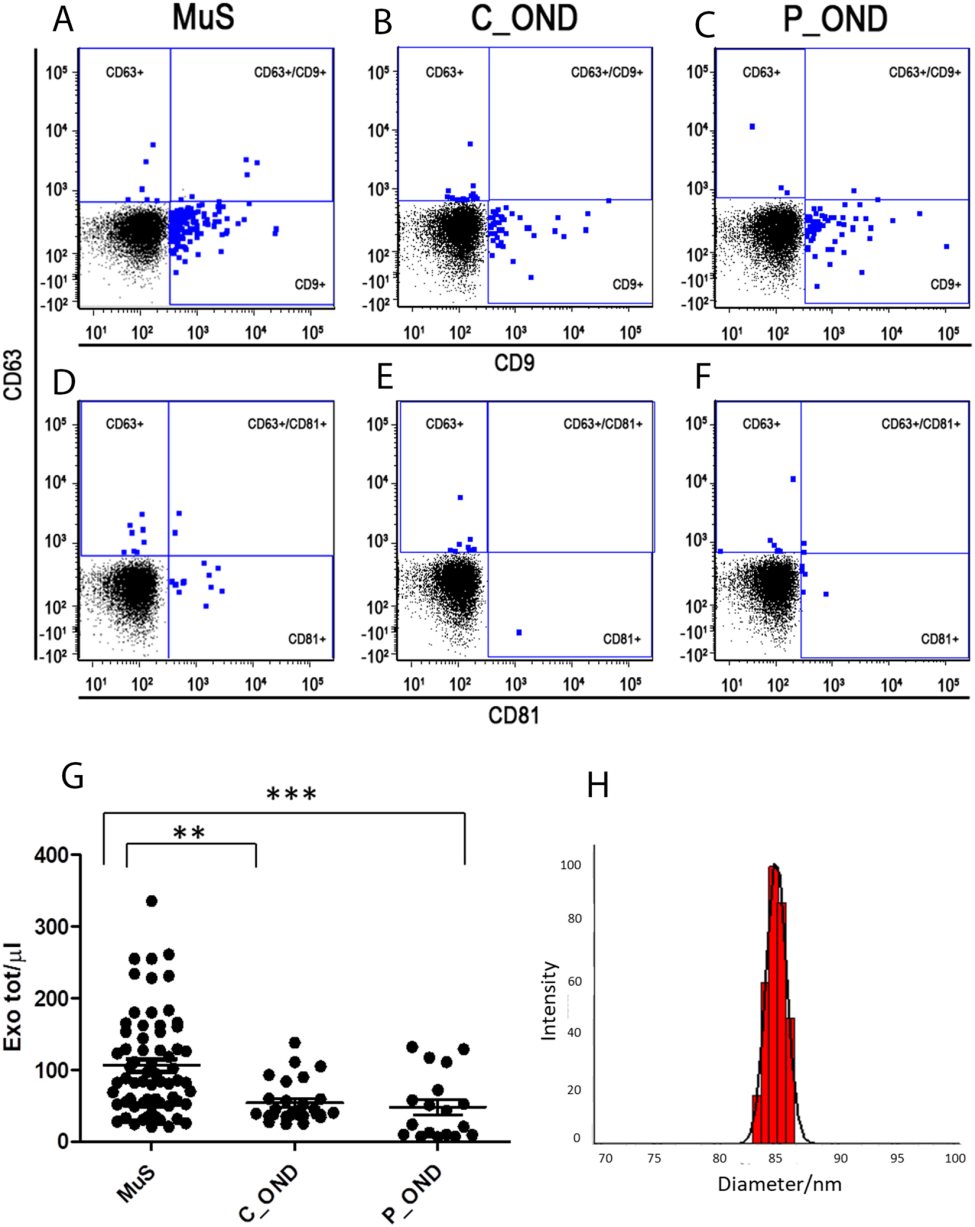
Panels (A–C) show the dot-plots of CD9 and CD63 surface expression for MuS, C_OND and P_OND CSF, respectively. Whereas, panels (D–F) show CD63 and CD81 positive exosomes from MuS, C_OND and P_OND CSF, respectively. (**G**) Dots plot shows the numbers of exosomes/μL gated in CSF of MuS patients compared with both OND clinical groups. (**H**) Hydrodynamic radius distribution of exosomes as obtained by Dynamic Laser Light Scattering analyses in terms of intensity distribution. **Means p < 0.001 and ***means p < 0.0001 of Kruskal-Wallis test with Dunn’s Multiple Comparison post-test.

ASMase is described to be matured in secretory isoform by Golgi and translocated into extracellular compartment by a still unclear mechanism^16^. To evaluate the potential relationship between the number of released exosomes in CSF and ASMase activity in MuS, a flow cytometry investigation was carried out. In Fig. 4 are reported representative flow cytometry data related to the exosomes content in the analyzed CSF, on the basis of their surface expression of tetraspanins Fig. 4 Panels A-C show the dot-plots of CD9 and CD63 surface expression for MuS, C_OND and P_OND CSF, respectively. Whereas, panels D-F show CD63 and CD81 positive exosomes from MuS, C_OND and P_OND CSF, respectively. Moreover, we analyzed a subgroup of patients by staining CD9, CD63 and CD81, as well as phosphatidylserin by PerCP-conjugated Annexin-V (BD Biosciences), showing no Annexin-V+ exosomes in our samples, as reported in Fig. S4. Based on this operative scheme, were identified, gated and counted the total exosomes in CSF from 66 MuS patients and 46 OND patients (depending to CSF availability, excluded outliers and divided into 28 C_OND and 18 P_OND), finding significantly high numbers of exosomes in MuS compared with both OND clinical groups (p < 0.001 for the comparison between MuS and C_OND and p < 0.0001 for the comparison between MuS and P_OND, Kruskal-Wallis test), as reported in Fig. 4G. The presence of exosomes was confirmed by dynamic laser light scattering experiments, where exosomes (mean diameter for the major population of 84 ± 7 nm) were detected. In particular the population is relatively homogenous as the polydispersivity index is 0.169 ± 0.07 (see Fig. 4H).

**Figure 5:**
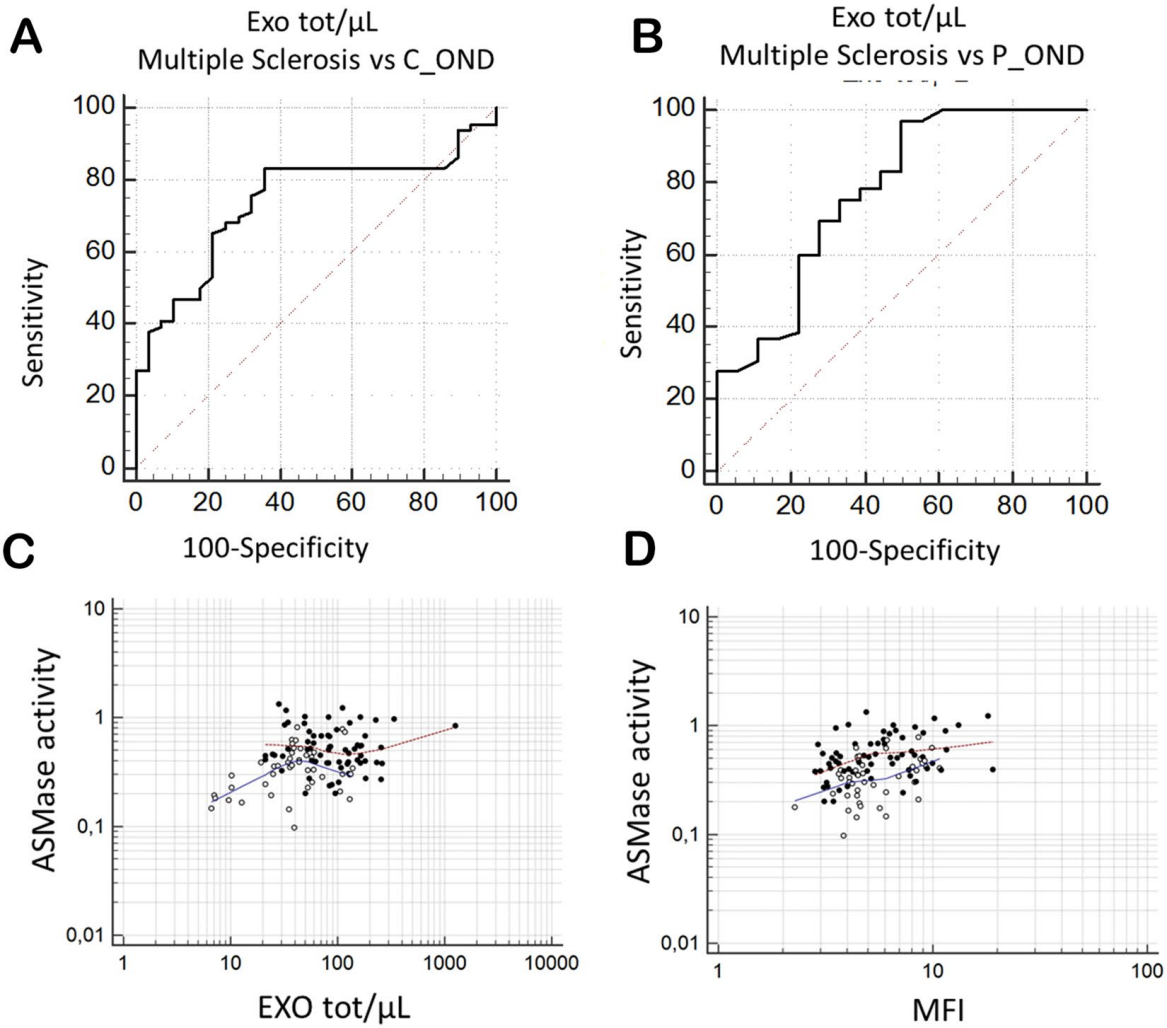
(**A**) ROC curve for number of Exosome/μL in MuS patients vs C_OND obtaining (p < 0.0001) AUC = 0.74, sensitivity = 83,33% and specificity = 64,29% for a cutoff = 45 exosomes/µl. (**B**) ROC curve for number of Exosome/μL in MuS patients vs P_OND obtaining AUC of 0.77 for a cut off = 58 exosomes/µl (sensitivity = 69.23% and specificity = 72.22%). (**C**) Significant Rank correlation (p < 0.001, r = 0,323) between ASMase activity and Exosome/μL gated in the entire patient case studies (109 patients divided into 65 MuS and 44 OND). Full dots are associated to MuS samples and empty dots for OND patients. (**D**) Significant Rank correlation (p < 0.001, r = 0,360) between the ASMase amount per exosome (MFI) with the measurement of ASMase activity by using the same 109 patients.

The high number of exosomes/µL was able to reclassify MuS from C_OND whit 83,33% sensitivity and 64,29% specificity, resulting in AUC = 0.74 (p < 0.0001) setting a cut off = 45 exosomes/µL (Fig. 5A). By comparing MuS vs P_OND (Fig. 5B) we obtained an AUC of 0.77 for a cut off = 58 exosomes/µL (sensitivity = 69.23% and specificity = 72.22%). Furthermore, our data show that the number of exosomes released in CSF is correlated (p < 0.001, r = 0,323) to ASMase activity measured in 109 patients, as reported in Fig. 5C, where full dots indicate MuS samples and empty dots indicate OND patients. These data demonstrate that both CSF ASMase activity and number of CSF exosomes are able to reclassify MuS subjects. Details of ROC curves and correlations are given in Supplementary Tables S5 and S6.

### Exosomes deliver active acid sphingomyelinase cargo

To better understand the relationship between exosomes and ASMase activity in CSF, we labeled the enzyme on these nano-vesicles with a fluorescent antibody. Figure 5D shows the correlation between the ASMase amount per exosome (MFI) with the measurement of ASMase activity by using the same 109 patients (MuS and OND) and demonstrating a correlation among the two parameters (p < 0.001, r = 0,360), without an evident separation between the analyzed groups. In order to verify the relationship between the number of exosomes and ASMase activity we measured no ASMase activity in CSF exosomes free fraction, as reported in Fig. S5. All together, these data demonstrate that CSF exosomes seem to be the nano-carriers of secreted ASMase, and probably, they generate a specific micro-environment to permit enzymatic activity versus endogenous SMs. Therefore, the activity is deeply influenced by the amount of enzyme delivered through exosomes, independently of the disease. Meanwhile, our data show (Supplementary Fig. S6 and Table S6) that the amount of ASMase per exosome is well correlating to Expanded Disability Status Scale (EDSS) registered for patients at the time of lumbar puncture (P < 0.05, r = 0.335 for Spearman test), indicating as the ASMase carried through extracellular vesicles may play important role in disease course.

## Discussion

MuS is presumed to be an autoimmune disease directed against the lipid-rich myelin sheath. We showed, through lipidomics approach, decreased SMs content in MuS CSF able to discriminate disease with good statistical performances. Moreover, our data suggest as level of SMs may serve as potential biomarkers. Further investigation demonstrated that low levels of SMs are due to the overexpression of active ASMase carried by specific exosomes. Actually, SMs are widely distributed in the nervous tissue and they are integral component of bio membranes, involved in many cellular function, including cell proliferation, signalling cascades and apoptosis^6^. Between sphingolipids, the SMs are the most abundant structural component of myelin and demonstrated that in adrenoleukodystrophy a depletion of SMs can be observed in demyelination areas, in accordance with our data^11,24^. The SMs are hydrolysed to ceramide by SMases, a family of enzymes strongly dependent on environmental pH. Ceramide serves as a potential lipid second messenger or mediator molecule modulating diverse cellular signal pathways e.g. apoptosis. Recently, patients with MuS were found to have increased CSF levels of ceramide C16:0 and C24:0 and these ceramide species were sufficient to induce neuronal mitochondrial dysfunction and axonal damage^12^. This evidence may be correlated with high degradation of SMs consistent with our data. Between these SMs, we found low level of C16:0 precursor (SM (d18:1/16:0)) in MuS in respect to OND.

To explain these results, we found overexpression of ASMase accompanied by high enzymatic activity in MuS CSF. Recently, ASMase was proposed as a novel target for the therapy of human autoimmune diseases, since it shows a pivotal role in regulation of human CD4 + T-cell activation and responses. In fact, several studies have shown that upon stimulation of CD3 and/or CD28, ASM-dependent ceramide signalling mediates intracellular downstream signal cascades of CD3 and CD28, and regulates CD4 + T-cell activation and proliferation^25^. Therefore, CD39^26^ and CD161^27^ have direct interactions with ASMase, which mediates downstream signals including STAT3 and mTOR and thus defines human Th17 cells.

**Figure 6:**
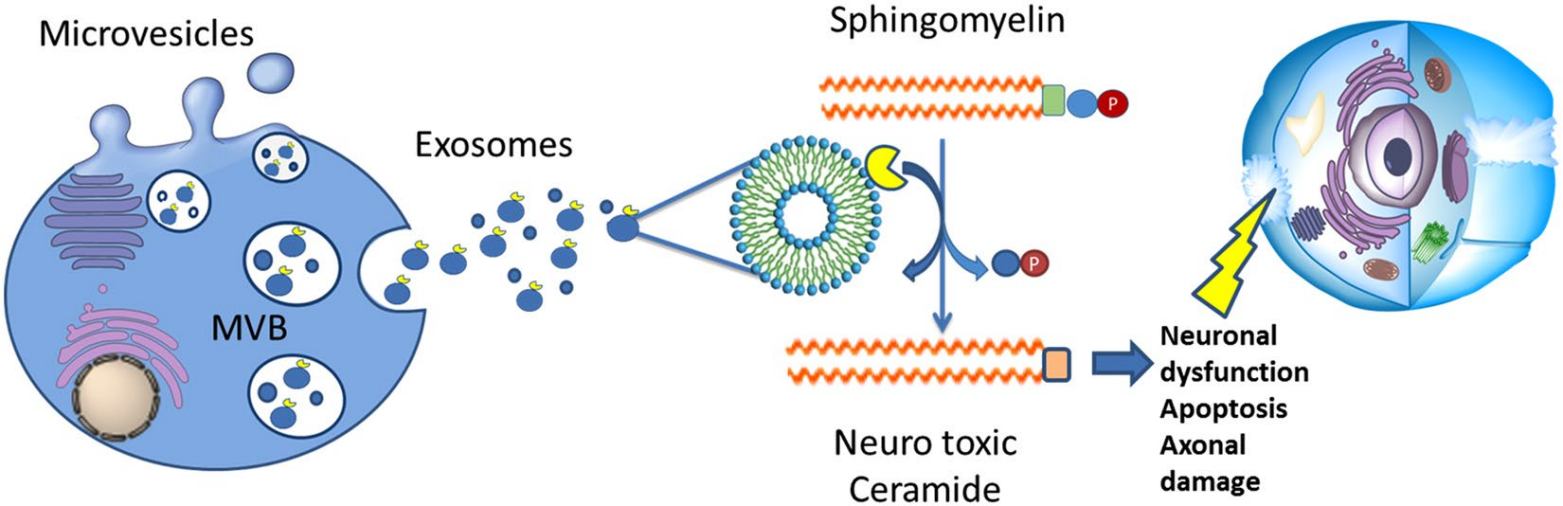
Proposed exosomal ASMase release and activity. The high number of released exosomes carry a cargo of active ASMase, which probably acts far from the original cells. The activity of the enzyme allows the conversion of SMs in Ceramides. Ceramides are neurotoxic messengers for cells. In particular ceramide is able to generate apoptosis, neuronal mitochondrial dysfunction and axonal damage. MVB = Multi-Vesicular Bodies.

In order to investigate whether the acid isoform of the enzyme may be active in the extracellular compartment, we studied exosomes related to ASMase cargo by flow cytometry. In fact, it is important to emphasize that exosomes effectively separate and protect their contents from the extracellular environment allowing intact cargo to be safely transported^28^. In addition, it has been demonstrated that the activation of ASMase is associated with MV release from glial cells, opening new strategies for the treatment of neuro-inflammatory disease^29^, even if we did not find data on ASMase as exosomal cargo. The number of exosomes was already characterized in serum of MuS patients^30^ and recently, it was demonstrated that they carry transcriptome biomarkers^31^. In this work, we show that the number of CSF exosomes is significantly higher in MuS patients compared to patients with OND, and that this number is correlated to CSF ASMase activity, highlighting an implication of exosomes in release and safe transport of ASMase in extracellular environment. Interestingly, we found that ASMase is located in CSF exosomes by labelling the enzyme with fluorescent antibody, showing that the ASMase abundance per exosome is strongly correlated to its activity. ASMase activity was known to act only close to membrane cell domain^16^, which allows the correct enzymatic condition. Our data show, for the first time, a high number of released exosomes carrying a cargo of active ASMase, that probably acts far from the original cells, inducing neuronal mitochondrial dysfunction and axonal damage through the conversion of SMs in ceramides (Fig. 6). To support this hypothesis, we correlated the quantity of ASMase per exosome to Expanded Disability Status Scale (EDSS) of patients, at the time of lumbar puncture, finding significant statistical correlation (p < 0.005) between parameters. Actually, mice lacking ASMase had a significant increase in myelin recovery and a significantly higher oligodendrocyte cell count after 2 weeks remyelination compared to wildtype littermates^14^.

In conclusion this work shows for the first time low levels of SMs and high ASMase activity in CSF from MuS patients, meanwhile the activity of this enzyme was related to the number of exosomes and to the content of enzyme per exosome. Finally, we demonstrated that CSF exosomes seem to be nano-carriers of secreted ASMase and that the exosomal ASMase content is related to MuS severity. Therefore, we can speculate that exosomes can generate a specific micro-environment to allow enzymatic activity versus endogenous SMs. This evidence may contribute to an improved understanding of the molecular mechanisms involved in MuS as well as development of novel diagnostic and therapeutic strategies. Our findings were obtained in a comparison between MuS and other Neurologic Disorders putatively characterized by lower or similar inflammatory processes (like Chronic Inflammatory Demyelinating Polineuropathy). Futures studies may consider comparison with other, less common diseases, like Neuromylitis Optica with antiacquaporin antibodies or Neurosarcoidosis, where the inflammatory burden could be similar to MuS Patterns.

## Materials and Methods

### Ethics Statement

The study design was made following the guidelines for the local Ethics Committee that approved the study (n. 18 of 31 october 2013, protocol n. 176, Ethic committee of “G. d’Annunzio” University and ASL N.2 Lanciano-Vasto-Chieti, Italy), and conducted according to Declaration of Helsinki (World Medical Association, 1997). All patients were informed about the procedures and provided written informed consent to participate in the study. In order to protect human subject identity a number code was employed for specimen identification.

Table 1 summarizes clinical and demographic features of the enrolled patients. All patients were selected in order to obtain age, sex and ethnicity (Caucasian people) matched cohorts of subjects. Patients with other disease course and comorbidites were excluded. CSF samples were collected at the MuS Center of Chieti (Italy) (Details are reported in Supplementary methods section).

**Table 1:** Demographical and Clinical features of enrolled patients.

	Multiple Sclerosis	C_OND	P_OND
Number of patients	95	45	31
Age range (mean ± SD)	16–61 (37 ± 10)	19–62 (43 ± 13)	39–92 (58 ± 13)
Gender (Female%)	58%	65%	42%
Drawing disease phase (% of patients)	90.5% Active	—	—
	9.5% Stable		
Diagnosis (% of patients)	94% RRMS	82.4% Cerebrovascular Disorder/Ischaemic Stroke (with white matter MRI hyperintensities-Leucoaraiosis = 75.8%)	77.4% CIDP
	3.1% CIS	11% Neurodegenerative disorders	3.2% Non-specific Diplopia
	3% Primary chronic-progressive	6.6% Meningoencephalits	12.9% Guillain–Barré syndrome
			3.2% Leber
MR data (% of patients)	12.6% Active	20% Atypica, GD negative	not performed
	3.1% Atypical, GD negative	2.2% Stable	
	45.3% Typical, GD negative	78% not performed	
	12.6% Typical, GD positive		
	3.1% Typical, GD not performed		
	23.3% MR not performed		
Link index (NV < 0,66) Range (mean ± SD)	0.44–2.83 (0.81 ± 0.43)	8.8% Regular	32.25% 0.29–0.58 (0.44 ± 0.07)
		28.8% 0.35–0.6 (0.47 ± 0.07)	67.7% not available
		62.4% not available	
BOC Pattern (% of patients)	74.7% Positive	6.6% Positive	3.2% Positive
	7.3% Negative	26.6% Negative	19.3% Negative
	18% not available	66.8% not available	77.5% not available
Barrier index (NV < 5.5) Range (mean ± SD)	2.8–15 (5.65 ± 2.44)	8.8% Regular	32.2% 3.9–15–1 (9.26 ± 3.05)
		28.8% 4.11–9.8 (5.79 ± 2.11)	67.7% not available
		62.4% not available	
EDSS at the time of lumbar puncture (% of patients)	7.5% EDSS = 0	—	—
	25% EDSS = 1–1.5		
	28.7% EDSS = 2–2.5		
	22.5% EDSS = 3–3.5		
	11.2% EDSS = 4–4.5		
	5% EDSS = 5–6		
Total Protein (µg/µL) Range (mean ± SD)	0.11–1.45 (0,36 ± 0.17)	0.11–1.11 (0.41 ± 0.20)	0.10–2.08 (0.59 ± 0.39)

C_OND = Other Central Neurological Disease; P_OND = Other Peripheral Neurological Disease; SD = Standard Deviation; RRMS = Relapsing-Remitting Multiple Sclerosis; CIS = Clinically Isolated Syndrome; MR = Magnetic Resonance; GD negative = absence of Gadolinium enhancement; GD positive = presence of Gadolinium enhancement; Typical = indicates a Brain MR suggestive of Multiple Sclerosis, Atypical = indicates a Brain MR not suggestive of Multiple Sclerosis; Regular = indicates a value of Link or Barrier Index inferior the normal value (NV); EDSS = Expanded Disability Status Scale; BOC = Oligo Clonal Bands. CIDP = Chronic Inflammatory Demyelinating Polineuropathy.

### LC-MS/MS lipidomics

A training set of samples (20 MuS and 17 OND) was used for the untargeted lipidomics approach. The extraction procedure was carried out on 200 µL of CSF as reported in our previous work^21^ and in Supplementary methods.

An HPLC Alliance HT 2795 Separations Module coupled to Quattro Ultima Pt ESI tandem quadrupole mass spectrometer (Waters Corporation, Milford, MA, USA) was used for lipidomics study. The total lipid extract was injected (20 µL) and analyzed following our already reported method with few modifications concerning the profile of biological phospholipids analyzed that was focused on the acquisition of SMs and Phosphatidylcholines (PCs)^22^. The differential lipid signals were tentatively identified by Lipidmaps Database and HMDB database, by the retention time and by using the mass fragmentation data.

### SDS-PAGE and Western blot

15 CSF from MuS patients and 15 CSF from OND subjects were used for SDS-PAGE and WB analysis (Example of raw WB performed was reported in Supplementary Fig. S7). CSF ASMase levels were analyzed by Western blot. Proteins were quantified by Bradford assay (Bio-Rad, München, Germany). Detail are reported in Supplementary methods.

### Enzymatic activity assay of sphingomyelinase

CSF from 87 MuS patients and CSF from 57 OND subjects were studied for ASMase and nSMase activity experiments by using the fluorescent substrate BODIPY-FL-C12-SM (N-(4,4-difluoro-5,7- dimethyl-4-bora-3a,4a-diaza-s-indacene-3-dodecanoyl) sphingosyl-Phosphocholine (ThermoFisher scientific). 6 μL of CSF were incubated following the protocols suggested by Mühle *et al*.^23^ for both acid and neutral isoforms. After incubation, the enzyme reaction was stopped at 100 °C for 10 minutes, samples were centrifuged for 15 minutes at 20880 g and finally transferred into polypropylene vial (Waters Corporation, Milford, MA, USA) for HPLC measurement of BODIPY-FL-C12-SM as substrate and BODIPY-FL-C12-CERAMIDE as product of the enzymatic activity. A Kinetex 5 µ XB-C18 100 Å 50 × 2.10 mm (Phenomenex, P/N 00B-4605-AN, S/N 639249-6) column fitted with guard column C18 4 × 2.0 mm (Phenomenex, P/N AJ0-4286) was used, kept at 45 °C. Analytes were monitored at emission wavelength 520 nm (excitation wavelength 488 nm, FLD). The enzyme activity was determined as a ratio of peak area of BODIPY-FL-C12-CERAMIDE (product) and the sum of peak area of the product and the BODIPY-FL-C12-SM (substrate). Data were normalized on the total proteins content for each CSF sample. Examples of chromatograms are reported in Fig. S8 in Supplementary materials.

### Exosomes staining for flow cytometry

From each enrolled subject, 100 µL of CSF were processed by a common flow cytometry no-lyse and no-wash method^32^. Briefly, CSF samples were stained using CD81-FITC (1 µl, clone: JS-81, CAT: 561956, BD Biosciences), CD63-PE (5 µl, clone: H5C6, CAT: 558020, BD Biosciences), CD9-BV450 (3 µl, clone: M-L13, CAT: 658167, BD Biosciences). After 30 min of staining (4 °C in the dark), 200 µL of PBS 1× were added to each tube and 1 × 10^6^ events/sample were acquired by flow cytometry (FACSVerse, BD Biosciences - three laser, eight color configuration, or FACSCanto, BD Biosciences - three laser, eight color configuration). In a separate tube, ASMase antibody (1 µl/test, 30 min at 4 °C in the dark) was added, using its specific secondary antibody FITC-conjugated (1 µl/test, Jackson Immuno Research, Cat. 115-095-146) after a dilution step (50 µl/test). Amplifier settings for forward scatter (FSC) and side scatter (SSC) as well as for any fluorescence channel were set in height (H) and in logarithmic mode. Exosomes scatter properties were established by running Megamix-Plus SSC beads (Biocytex, Marseille, France) at the same photomultiplier (PMT) voltages used for exosome detection. Each antibody/reagent was titrated (8 point titration) under assay conditions; dilutions were established based on achieving the highest signal (mean fluorescence intensity, MFI) for the positive population and the lowest signal for the negative population, representing the optimal signal to noise ratio^33^, and stain indexes were calculated^34^. The immune complex formation and unspecific background linked to antibody aggregation was prevented by spinning the antibody stock solution before use, at 21,000 g for 12 minutes.

Instrument performances, data reproducibility and fluorescence calibrations were sustained and checked by the Cytometer Setup & Tracking Beads (BD Biosciences). In order to evaluate non-specific fluorescence, Fluorescence Minus One (FMO) controls were used^35^. Compensation was assessed using CompBeads and FACSuite FC Beads (BD Biosciences) and single stained fluorescent samples. Data were analyzed using FACSuite v 1.0.5 (BD Biosciences) and FCS Express 5.01 software (De Novo Software, Glendale, CA). Exosome numbers were obtained by volumetric count.

#### Gating strategy

A morphological gate was placed on a SSC-H/FSC-H dot-plot on the basis of the area containing the exosome compartment established by running MegMix-Plus SSC Beads (Biocytex, Marseille, France). The exosome compartment was analyzed for the expression of CD9, CD63 and CD81 and Annexin V (in a sub-group of patients) on the basis of the respective FMO control, while the ASMase compartment was identified on the basis of the respective negative control (unspecific bound of the specific secondary antibody on the exosome gate). MFI Ratio for the ASMase was calculated by dividing the MFI of the positive events by the MFI of the secondary antibody alone^36^. Aggregates of exosomes were monitored by plotting both FSC-Area (FSC-A) signals versus FSC-High (FSC-H) signals, or SSC-Area (SSC-A) signals versus SSC-High (SSC-H). As evidenced in the representative dot-plots reported in Fig. S9, by analyzing non-manipulated samples, the majority of exosomes (more than 98%) are single events.

#### Exosome sorting

Exosomes were separated, by instrumental cell sorting (70 µm nozzle, FACSAria III, BD Biosciences), on the basis of the above described flow cytometry method and gating strategy. Pure samples (92–97% of purity) were analyzed for their size by Dynamic Light Scattering.

### Dynamic Laser Light Scattering

Pure exosomes dimensions have been determined through Dynamic laser Light Scattering by using a 90Plus/BI-MAS Zeta Plus multiangle particle size analyzer (Brookhaven Instruments Corp.) and showed in Table 2. The size of the exosomes has been calculated from the translational diffusion coefficient by using the Stokes-Einstein equation [d = k T/3μη D, where d is the particle diameter, k is the Boltzmann’s constant, T is the absolute temperature, *k* is the fluid viscosity, D is the translational diffusion coefficient]. Data have been analyzed in terms of both scattering intensity and particles number by using either a monomodal or a multimodal size distribution software depending on the specific sample. The polydispersity index has been used as a measure of the homogeneity of the exosomal population.

**Table 2:** Dynamic Laser Light Scattering Determination.

Diameter(Intensity)^a^		Diameter(Number)^b^	
N. Populations	Mean Population	N. Populations	Mean Population
1	84 ± 7	1	81.3 ± 9.7

^a^Data evaluated by using intensity distribution of the scattered light. ^b^Data evaluated by using number distribution of particles monitored.

### Statistical Analysis

Mass spectra obtained by LC-MS/MS phospholipids analysis were processed using MarkerLynx software (Waters, Milford, USA). After the exclusion of lipid signals absent in 75% of samples, that resulted the optimum compromise to eliminate most of the useless variables, data matrix was subjected to different explorative statistical approaches.

The first multivariate statistical analysis was performed using Neural Designer software (version 2.9.5) by neural network implementation on data matrix. Partial least squares Discriminant Analysis (PLS-DA), was also performed on the same data matrix, by using SIMCA-P + 11.0 software (Umetrics AB, Umea, Sweden). Lastly, the univariate statistical analysis visualized as Volcano-plot using Metaboanalyst 3.0 software, was carried out. The significant lipid signals resulted by comparing the above mentioned statistical approaches, were used to perform a combined ROC curve, an internal and external diagnostic model validations and a logistic regression model, using Metaboanalyst 3.0 software. Unpaired t test with Welch’s correction or Kruskal-Wallis test with Dunn’s Multiple Comparison post-test were performed for comparisons between clinical groups, using GraphPad Prism (GraphPad software, Inc. USA). The ROC curve analysis based on single variable, and Rank correlation between variables were performed using MedCalc 7.6 (MedCalc software bvba), by using a Spearman non-parametric correlation test. The values of p < 0.05 were considered significant. 95% of confidence interval was assumed for each test. ChemBioDraw Ultra 13.0 software was used for the cartoon making of the proposed mechanism reported in Fig. 6.

## Electronic supplementary material

Supplementary Materials
