# Supplementary material for: Enhanced release of acid sphingomyelinase-enriched exosomes generates a lipidomics signature in CSF of Multiple Sclerosis patients
Source: Sci Rep. 2018 Feb 15;8:3071. doi: 10.1038/s41598-018-21497-5 (PMC5814401; doi:10.1038/s41598-018-21497-5)
Supplement: Supplementary file 1 — Supplementary Materials [file 41598_2018_21497_MOESM1_ESM.docx]

**Supplementary material of:**

**Enhanced release of acid sphingomyelinase enriched-exosomes generates a lipidomics signature in CSF of multiple sclerosis patients**

Damiana Pieragostino, PhD^1,2^, Ilaria Cicalini, MSc^2,3^, Paola Lanuti, PhD^2,4^, Eva Ercolino, PhD^2,4^, Maria di Ioia, MD^5^, Mirco Zucchelli, PhD^2^, Romina Zappacosta, PhD^3^, Sebastiano Miscia, PhD^2,4^, Marco Marchisio, PhD^2,4^, Paolo Sacchetta, PhD^1,2^, Marco Onofrj, MD^2,5^and Piero Del Boccio, PhD^2,3^*

^1^Department of Medical, Oral and Biotechnological Sciences, University ‘‘G. d’Annunzio’’ of Chieti-Pescara, Chieti, Italy.

^2^Centre on Aging Sciences and Translational Medicine (Ce.S.I-MeT), University ‘‘G. d’Annunzio’’ of Chieti-Pescara, Chieti, Italy.

^3^Department of Pharmacy, University ‘‘G. d’Annunzio’’ of Chieti-Pescara, Chieti, Italy.

^4^Departmenton Medicine and Aging Sciences, “G. d’Annunzio” University of Chieti-Pescara, Chieti, Italy

^5^Department of Neuroscience, Imaging and Clinical Sciences, “G. d’Annunzio” University of Chieti-Pescara, Chieti, Italy

***Keywords:*** Lipidomics; CSF Exosomes; Acid Sphingomyelinase.

*Corresponding Author:

Prof. Piero Del Boccio, PhD
Department of Pharmacy, University ‘‘G. d’Annunzio’’ of Chieti-Pescara, Chieti, Italy.
[e-mail: piero.delboccio@unich.it](mailto:e-mail:%20p.delboccio@unich.it)

Phone: +39 0871 3554516

Fax: +39 0871 541598

**Supplementary Methods**

**Patients and Sample collection**

95 patients with MuS, in accordance with the 2010 Polman’s criteria(1), were included in this study. Clinical diagnosis was confirmed by MRI studies and by the presence of oligoclonal bands in CSF. The Expanded Disability Status Scale (EDSS) score was obtained at the time of lumbar puncture. To be enrolled in the study patients should have not been treated with steroids in the month before study entry and should have been never treated with immunomodulatory or immunosuppressive drugs both for MuS and for other diseases. 76 CSF samples of patients with other neurological diseases (OND), divided into 45 C_OND and 31 P_OND, were used as control groups. The diagnosis in each of these patients was defined according to individual disease diagnostic criteria.CSF samples, taken by a routine lumbar puncture at L3/L4 or L4/L5 interspace, were always collected in the morning on the first day of patient observation. Each sample (around 3 mL) was centrifuged at 10000g at 4°C, for 10 minutes. The supernatant was divided into aliquots and snap-frozen at -80°C. Around 2 ml of CSF from each subject was used for diagnosis. 400 microliters of CSF per patient were employed in this study (200 µL for untargeted lipidomics, 6 µL for the enzymatic activity, about 40 µL for WB experiments, 100 µL for flow cytometry study and 30 µL for protein quantification). The entire cohort of recruited patients was managed in various experiments depending on the amount of CSF available for each sample.

**Lipid extraction procedure**

Briefly, CSF samples were fortified by adding 10 μL of a solution of Lyso-Sphingomyelin 100 μg/mL. After vortexing, 300 μL of methanol was added for protein precipitation. The obtained solution was vortexed, gently shacked for 5 minutes at 10°C and centrifuged for 15 minutes at 20880 g (T=10°C). After centrifugation 1 mL of Methyl Tert-Butyl Ether (MTBE) and 250 μL of water were added to supernatant (350μL), vortexed and centrifuged at the same conditions. The upper MTBE phase was dried and finally dissolved in 100 μL of ACN, vortexed and centrifuged for 15 minutes at 20880 g (T=20°C). Ninety microliters of this solution were recovered and 40 μL was transferred in the vials for LC-MS/MS analysis. The remaining 50 μL were stored at -80°C for further analyses.

**SDS-PAGE and Western blot**

18 μg of protein samples were separated by 8% SDS-PAGE, transferred onto PVDF membranes, and blocked for 1 hours at room temperature using 10% dry milk dissolved in PBS-Tween 1%. After blocking, membranes were incubated with anti-rabbit ASMase antibody (1:300, Cell Signaling Technology) over night at 4°C. Next, membranes were washed with PBS-Tween and incubated with peroxidase-conjugated goat anti-rabbit (1:1000 Sigma-Aldrich St. Louis MO, USA) secondary antibody for 1 hour at room temperature. Visualization was achieved using the enhanced chemiluminescence method (SuperSignal West Dura Extended Duration Substrate, ThermoFisher scientific). For quantification of ASMase, the OD of the specific bands were measured and normalized to precursor isoform of ASMase (pro-ASMase) using the Abode Photoshop CS6 software.

**Table S1:** Confusion matrix obtained by neural network implementation on 421 lipid signals. The rows represent the target classes and the columns the output classes for the testing target data set. The decision threshold is 0.5. The number of correctly classified instances is 6, and the number of misclassified instances is 1.

|  | **Predicted positive** | **Predicted negative** |
| --- | --- | --- |
| Actual positive | 4 | 0 |
| Actual negative | 1 | 2 |

**Table S2:** A new sample prediction analysis on the testing set of patients (20%) obtained by the diagnostic power analysis performed on the training set of patients (80%). Table shows that five patients out of six are correctly diagnosed (0 means OND class, while 1 means MuS), with the associated probability.

| Name | Probability | Class |
| --- | --- | --- |
| OND_a | 0.73893 | 1 |
| OND_b | 0.96431 | 0 |
| OND_c | 0.5109 | 0 |
| MuS_a | 0.96361 | 1 |
| MuS_b | 0.63039 | 1 |
| MuS_c | 0.99092 | 1 |

**Table S3:** Logistic Regression Model. P is Pr(y=1|x). The best threshold (or Cutoff) for the predicted P is 0.45. Labels in Logistic Regression: 0/1.

(Case:1= MuS and Case:0=OND)

| **Logistic regression function** |
| --- |
| logit(P) = log(P / (1 - P)) = 4.626 - 0.059 SMd181/161 - 0.41 SMd181/24115Z - 0.658 SMd182/221 - 0.01 SMd180/1619ZOH - 0.812 SMd181/140 - 1.024 SMd182/200 - 0.067 SMd181/130 |

**Table S4:** Performance of Logistic Regression Model

|  | **AUC** | **Sensitivity** | **Specificity** |
| --- | --- | --- | --- |
| Training/Discovery | 0.955 (0.931 ~ 0.980) | 0.954 (0.921 ~ 0.987) | 0.873 (0.815 ~ 0.931) |
| 10-fold Cross-Validation | 0.702 (0.492 ~ 0.912) | 0.824 (0.824 ~ 1.000) | 0.786 (0.571 ~ 1.000) |

| Statistical Parameters | ROC_Fig. 3C | ROC_Fig. 3D | ROC_Fig. 5A | ROC_Fig. 5B |
| --- | --- | --- | --- | --- |
| Variable | ASMase  activity | ASMase activity | Exosomes tot/ µL | Exosomes tot/ µL |
| Sample size | 110 | 121 | 94 | 83 |
| Positive group | 87 Multiple Sclerosis (79,09%) | 87 Multiple Sclerosis (71,90%) | 66 Multiple Sclerosis(70,21%) | 65 Multiple Sclerosis (78,31%) |
| Negative group | 23 P_OND (20,91%) | 34 C_OND(28,10%) | 28 C_OND (29,79%) | 18 P_OND(21,69%) |
| Area under the ROC curve (AUC) | 0,88 | 0,68 | 0,74 | 0,77 |
| Standard Error ^a^ | 0,036 | 0,053 | 0,052 | 0,0687 |
| 95% Confidence interval ^b^ | 0,804 to 0,934 | 0,586 to 0,759 | 0,638 to 0,824 | 0,669 to 0,859 |
| z statistic | 10,374 | 3,313 | 4,532 | 3,988 |
| Significance level p | <0,0001 | <0,001 | <0,0001 | <0,0001 |
| Youden index J | 0,651 | 0,269 | 0,476 | 0,469 |
| Associated criterion | >0,34 | >0,43 | >45 | >58 |
| Sensitivity (%) | 78,16 | 56,32 | 83,33 | 69,23 |
| Specificity (%) | 86,96 | 70,59 | 64,29 | 72,22 |
| ^a^ DeLong et al. 1988 ^b^ Binomial Exact | | | | |

**Table S5: Statistical parameters of ROC curves**

**Table S6: Statistical Parameters of Correlations**

| Statistical Parameters | Correlation Fig. 5C | Correlation Fig. 5D | Correlation Fig. 5S |
| --- | --- | --- | --- |
| Variable Y | ASMase Activity (Log) | ASMase Activity (Log) | MFI |
| Variable X | Exosomes tot/ µL (Log) | MFI (Log) | EDSS |
| Sample size | 109 | 108 | 53 |
| Correlation coefficient r | 0,323 | 0,360 | 0,335 |
| Significance level | <0,001 | <0,001 | 0,013 |
| 95% Confidence interval for r | 0,1438 to 0,4819 | 0,1837 to 0,5141 | 0,07085 to 0,5548 |

**Figure S1**

**Figure S1**: A graphical representation of the final architecture obtained by the implementation of neural network statistical analysis. The yellow circles represent scaling neurons, the blue circles perceptron neurons and the red circles probabilistic neurons. The number of inputs (lipid signals) is 96, and the number of outputs (diagnosis) is 1. The complexity, represented by the numbers of hidden neurons, is 3.

**Figure S2:**

**Figure S2**: enzymatic activity (following the preparation protocol suggested by Mühle et al. as reported in the method section and in reference 23) of neutral SMase in CSF of pooled MuS patients’ vs pooled OND (divided in Central and Peripheral disease), by using fluorescent SM-FLBODIPY incubated with CSF and analyzed by HPLC-FLD. C_OND and P_OND mean Other Central and Peripheral Neurological Diseases, respectively. As highlighted in the box green, no reaction product (ceramide) was measured.

**Figure S3**

**
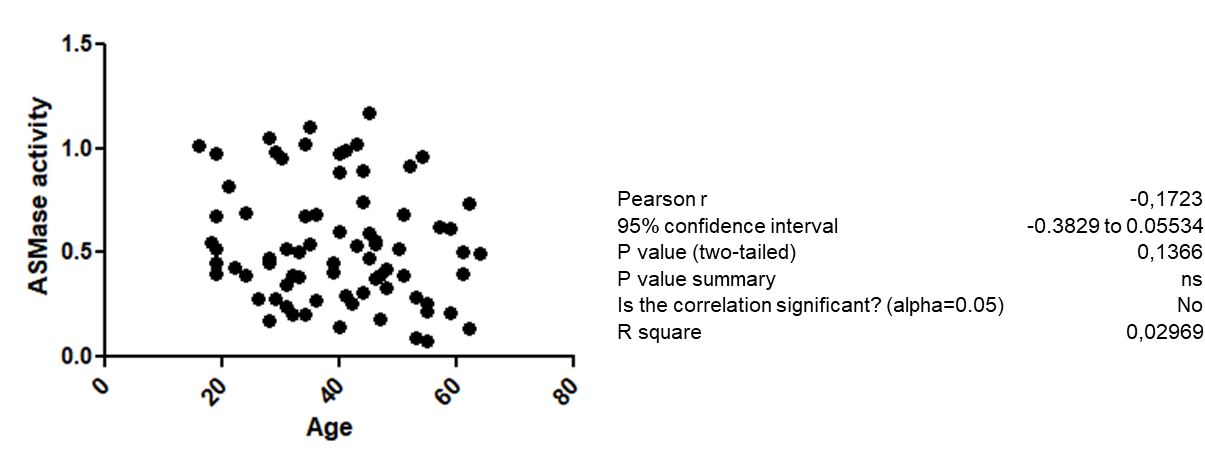
**

**Figure S3**: Spearman correlation test (p=0.13) between age and ASMase activity in CSF.

**Figure S4**

**Figure S4**: Exosomes were identified, and Annexin V binding was evaluated. As shown, no Annexin V+ exosomes have been found. Dot-plot is representative of 10 separate experiments.

**Figure S5**

**Figure S5:** Panels A-B show the dot-plots of Sphingo surface expression (detected on the FITC-H channel) for a pool of CSF (panel A) and the same pool after depletion of exosomes through ultracentrifugation (2) (panel B). Whereas, panels C-D show chromatograms obtained by FLD-HPLC analysis of ASMase activity. Red arrows show the peak of the BODIPY-FL-C12-Cer, obtained through enzymatic reaction. In particular Panel C shows BODIPY-FL-C12-SM incubated with CSF pool for 24h. Panel D shows BODIPY-FL-C12-SM incubated with the CSF exo free fraction. In panel E was reported the histogram of the activity of enzyme in the two conditions analyzed (CSF and exo free CSF). Experiment demonstrated the absence of activity in CSF pool without exosomes carrying ASMase.

**Figure S6**


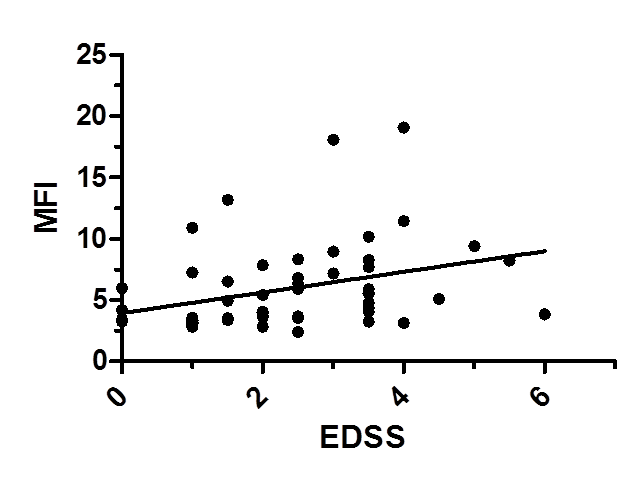


**Figure S6**: Significant Spearman correlation (p=0.01) between MFI and Expanded Disability Status Scale (EDSS) based on 53 MuS patients.

**Figure S7**


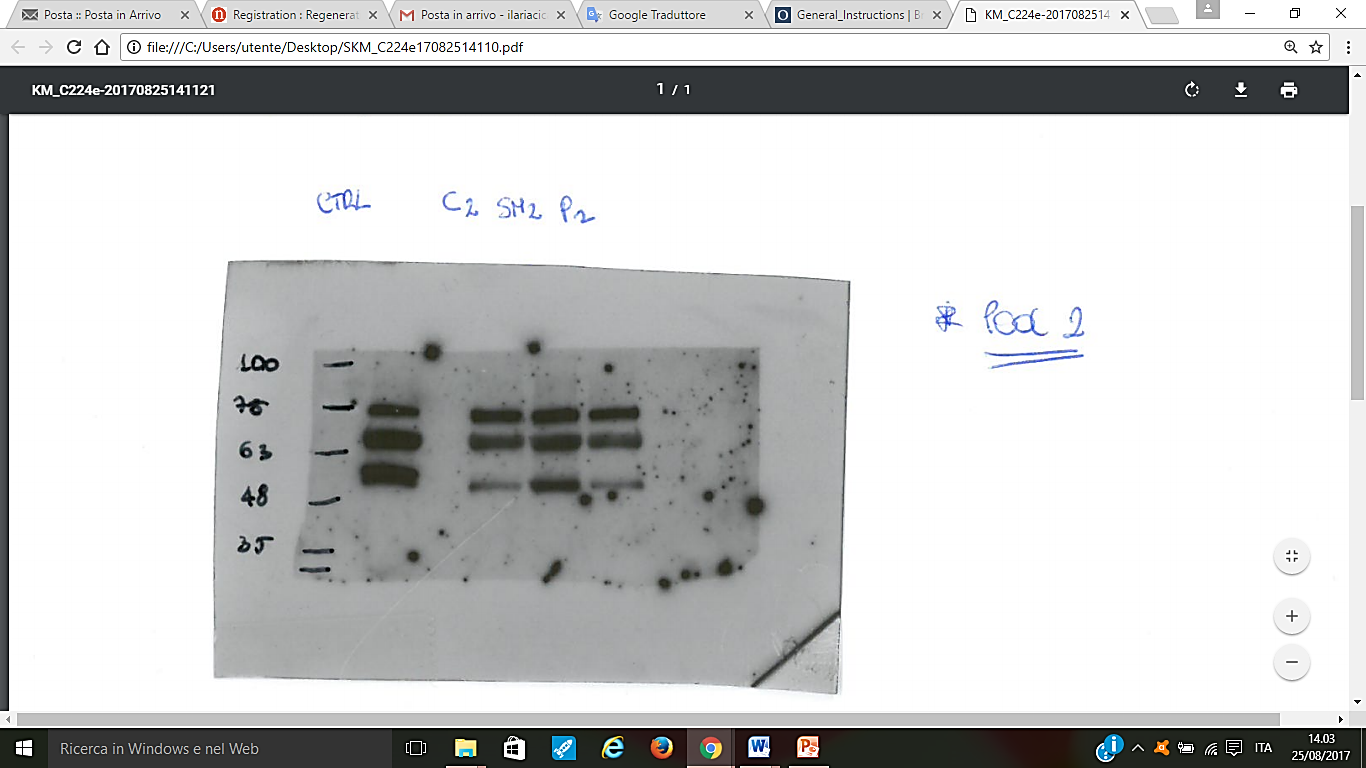


Positive control

MuS

C_OND

P_OND

Marker

100 KDa

75 KDa

63 KDa

48 KDa

35 KDa

**Figure S7:** Raw WB of the Fig.2experiment. This WB experiment was useful to evaluate the expression of ASMase in MuS patients versus Other Central and Peripheral Neurological disease (C_OND and P_OND, respectively).

**Figure S8**


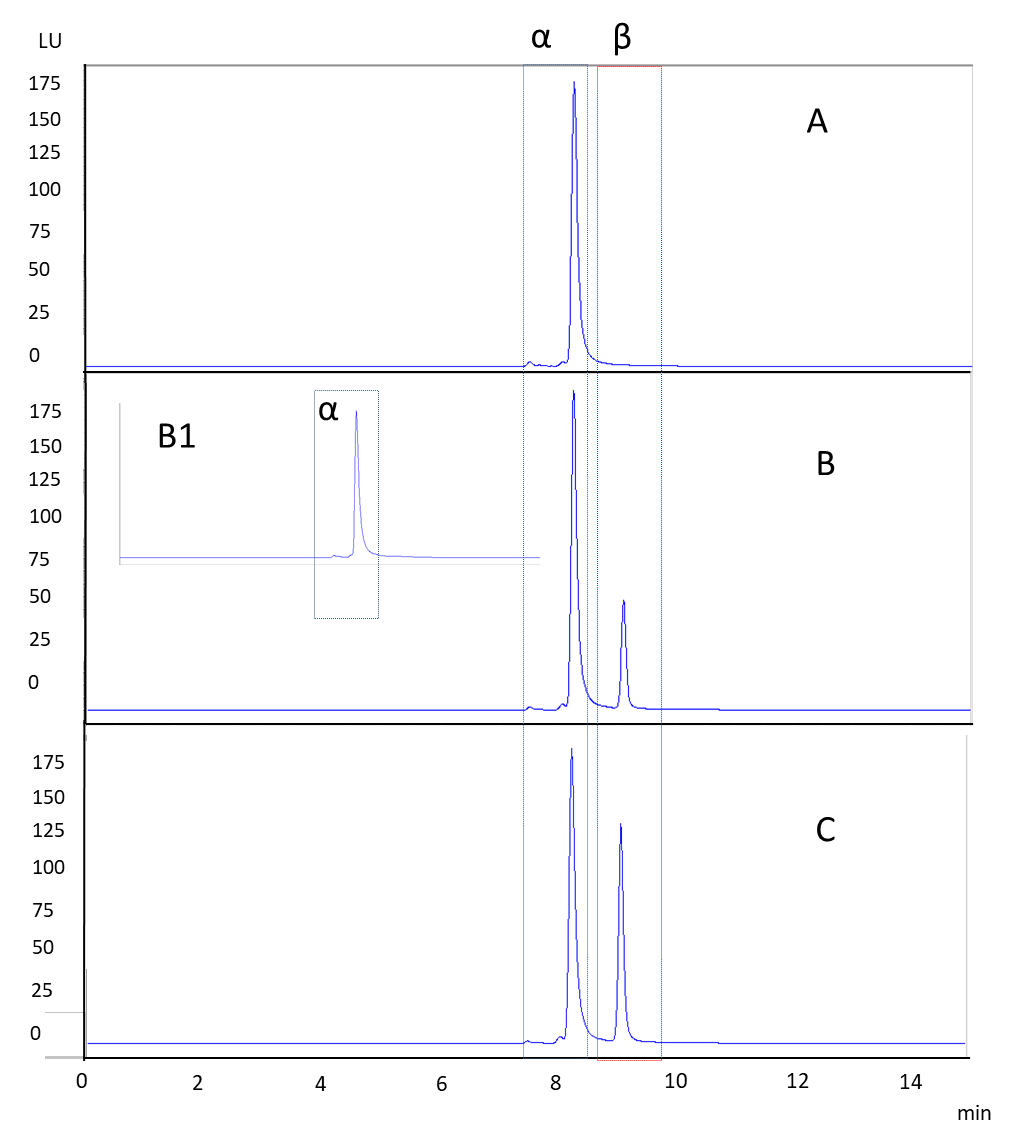


**Figure S8**: Figure shows chromatograms obtained by FLD-HPLC analysis of ASMase activity. Blue α section shows the peak of BODIPY-FL-C12-SM (N-(4,4-difluoro-5,7- dimethyl-4-bora-3a,4a-diaza-s-indacene-3-dodecanoyl), while red βsection shows the BODIPY-FL-C12-Cer, obtained through enzymatic reaction. Panel A: BODIPY-FL-C12-SM with CSF, without incubation. Panel B and C show BODIPY-FL-C12-SM incubated with CSF for 24h and 48h respectively. Panel B1: BODIPY-FL-C12-SM without CSF, incubated for 24h. The HPLC setup consisted in an Agilent 1100 series autosampler (G1329A), column oven (G1316A), degasser (G1322A), quaternary pump (G1311A), FLD (G1321A) and DAD (G1315B) detectors. Instrumental control, data acquisition, and processing were all carried out using ChemStation software (Agilent Technologies, Wokingam, UK).The mobile phase consisted of a gradient mix of water 0.1% trifluoracetic acid (A) and acetonitrile 0.1% trifluoroacetic acid (B). The injection volume of 50 μL was used for all analyzed samples. Analytes were identified on the basis of their retention time: BODIPY-FL-C12 at 7.1 min, BODIPY-FL-C12-SM (ThermoFisher scientific) at 8.3 min and BODIPY-FL-C12-CERAMIDE at 9.1 min. A working solution containing 0.1 μg/ml of BODIPY-FL-C12 and BODIPY-FL-C12-SM was analyzed during the entire analytical section to test the reproducibility of the method.

**Figure S9**

**Figure S9**: Aggregates of exosomes were monitored by plotting both FSC-Area (FSC-A) signals versus FSC-High (FSC-H) signals, or SSC-Area (SSC-A) signals versus SSC-High(SSC-H). As evidenced in the representative dot-plots, by analyzing non-manipulated samples, the majority of exosomes (more than 98 %) are single events.

**References**

1. Polman CH*, et al.* (2011) Diagnostic criteria for multiple sclerosis: 2010 revisions to the McDonald criteria. *Annals of neurology* 69(2):292-302.

2. Théry C, Amigorena S, Raposo G, Clayton A.(2006) Isolation and characterization of exosomes from cell culture supernatants and biological fluids. Curr Protoc Cell Biol. 2006 Apr;Chapter 3:Unit 3.22.
